# Current Approaches on Viral Infection: Proteomics and Functional Validations

**DOI:** 10.3389/fmicb.2012.00393

**Published:** 2012-11-16

**Authors:** Jie Zheng, Boon Huan Tan, Richard Sugrue, Kai Tang

**Affiliations:** ^1^Division of Chemical Biology and Biotechnology, School of Biological Sciences, Nanyang Technological UniversitySingapore; ^2^Defence Medical and Environmental Research Institute, DSO National LaboratoriesSingapore; ^3^Division of Molecular and Cell Biology, School of Biological Science, Nanyang Technological UniversitySingapore

**Keywords:** virus infection, host responses, virus-host interactions, activity-based functional validations, mass spectrometry based proteomics

## Abstract

Viruses could manipulate cellular machinery to ensure their continuous survival and thus become parasites of living organisms. Delineation of sophisticated host responses upon virus infection is a challenging task. It lies in identifying the repertoire of host factors actively involved in the viral infectious cycle and characterizing host responses qualitatively and quantitatively during viral pathogenesis. Mass spectrometry based proteomics could be used to efficiently study pathogen-host interactions and virus-hijacked cellular signaling pathways. Moreover, direct host and viral responses upon infection could be further investigated by activity-based functional validation studies. These approaches involve drug inhibition of secretory pathway, immunofluorescence staining, dominant negative mutant of protein target, real-time PCR, small interfering siRNA-mediated knockdown, and molecular cloning studies. In this way, functional validation could gain novel insights into the high-content proteomic dataset in an unbiased and comprehensive way.

## Introduction

Invasive viruses are adaptively infectious and pathogenic. Although host cells evolve and occupy a network of multiple defensive measures, microbial pathogens could in turn manipulate cellular machinery to counteract those immune defenses in order to evade or neutralize them (Finlay and McFadden, 2006). This might be in part attributed to the fact that viruses evolve and mutate much more quickly than their hosts, and result in emerging mutants with enhanced viral attacks. For instance, viral genomes of RNA viruses, e.g., influenza A virus, intrinsically exhibit hyper-variations and continued mutations due to a lack of proofreading RNA dependent RNA polymerases (Holland et al., 1982). This may also be derived from the diversity of virus families and a vast number of formidable viruses threatening mankind; for examples, the representatives are human immunodeficiency virus, influenza virus, respiratory syncytial virus (RSV), severe acute respiratory syndrome (SARS), dengue virus, and so on. Epidemic or even pandemic diseases occurred in recent years upon breakout of those infectious viruses.

In recent decades, the field of virology is rapidly expanding with the advances of high throughput genome sequencing and proteome screening technologies. To date, the numbers of complete viral genomes and proteomes that cover 118 taxonomy groups have reached to 2853 and 1932 in the NCBI and Uniprot databases, respectively^1^^,^^2^. As these databases expand, the daunting challenge still lies in illustrative delineation of sophisticated pathogen-host interactions or virus-hijacked signaling pathways. Advancements in mass spectrometry (MS) based proteomics have tremendously facilitated the investigations of viral proteomes as well as host responses associated with viral infections. Several popular MS based approaches have been applied to study viruses and their hosts (Table 1). These include 2D gel, tandem affinity purification (TAP), co-immunoprecipitation (Co-IP), and quantitative stable isotope labeling strategies, such as isotope coded affinity tag (ICAT), isobaric tag for relative and absolute quantitation (iTRAQ), and stable isotope labeling of amino acids in cell culture (SILAC). Others developed novel approaches such as HLA peptidome scanning chips to study host responses upon virus infection (Zheng et al., 2011). These MS based proteomic approaches are capable to explore from individual binding partners to quantitatively altered proteomes upon virus infections. Moreover, two other MS based approaches developed to study protein–protein interactions (PPIs) and protein conformational changes have also contributed to virology studies. These are chemical cross-linking and hydrogen/deuterium exchange (HDX) based MS methodologies.

**Table 1:** **A summary of proteomic techniques and their applications in virology**.

Mass spectrometry based proteomic approaches	Main applications in virology	Relevant reference
Tandem affinity purification based MS approach	Protein–protein interactions	Jorba et al. (2008), Mayer et al. (2005), Mayer et al. (2007)
Co-immunoprecipitation based MS approach	Binding partners of target protein	Moresco et al. (2010), Noisakran et al. (2008)
HLA peptidome scanning chip based MS approach	Searching for disease related peptides or differently expressed proteins after virus infection	Herberts et al. (2003), Wahl et al. (2010)
Isotope coded affinity tag (ICAT)		Yan et al. (2004), Booy et al. (2005)
Isobaric tag for relative and absolute quantitation (iTRAQ) Stable isotope labeling of amino acids in cell culture (SILAC) Difference gel electrophoresis (DIGE)	Mapping differently expressed proteins in host proteome upon virus infection	Zhang et al. (2009), Chen et al. (2008) Dreger et al. (2009), Hammond et al. (2010) Pastorino et al. (2009), Sun et al. (2011a)
Cross-linking based MS approach	Investigating protein-nucleotides binding sites	Deval et al. (2007), Bhardwaj et al. (2008)
Hydrogen/deuterium exchange (HDX) based MS approach etc.	Conformational dynamics of host or viral proteins	Lisal et al. (2005), Monroe et al. (2010)

## Cross-Linking Based MS Methodology

Formaldehyde cross-linking could be applied to study the binding between nucleotides, e.g., DNA and RNA, and proteins (Petrotchenko and Borchers, 2010). Formaldehyde is a reactive cross-linking agent that could bind nucleic acids, peptides, or proteins. Its carbon atom center is nucleophilic to bind cytosine and it could also react with side chains of lysine, arginine, histidine, and cysteine to form methylol groups, schiff-bases, or methylene bridges. These cross-links could be reversed by heating and the eluted peptides are further submitted to MS for sequence identifications (Orlando et al., 1997; Metz et al., 2004). For its applications, the helicase-like regions within the viral polymerase involved in RNA binding were characterized by reversible formaldehyde cross-linking and MS (Kim et al., 2005; Deval et al., 2007; Han et al., 2009).

## Hydrogen/Deuterium Exchange Based MS Methodology

Hydrogen/deuterium exchange combined with MS is able to investigate the protein structures and dynamics by studying their conformational alternations. Some amide hydrogens at the backbone could be readily exchanged when incubated in a deuterated environment while some amide hydrogens hidden in the interior of the protein or involved in hydrogen bonding have restricted access to deuterium. A quench condition (0°C, pH 2.5) is used to stop the exchange reaction followed by pepsin digestion of proteins prior to MS analysis. Therefore, the deuterium labeling induced mass shift and H/D exchange rate could reflect the protein conformational information and hydrogen bond interactions (Hamuro et al., 2003; Engen, 2009). HDX combined with high resolution MS was capable of studying structural information such as protein-nucleic acid bindings, protein–protein interplays, and protein maturation rearrangements. Viral molecular motors, e.g., helicases or packaging factors, are associated with nucleic acid binding and hydrolysis functions. The hexameric packaging motor (P4) of cystovirus enabled to bind viral RNAs through its RNA binding channel coupled with ATPase activities. The conformational dynamics of P4 in the presence and absence of RNA were examined by HDX coupled with MS. The HDX kinetics revealed distinctive states for different domains of P4 in response to nucleotide-binding, RNA loading, and translocation as well as ATPase activities, and thus provided a comprehensive understanding to P4 molecular architecture in different biological states (Lisal et al., 2005). Assembly of MS2 viral coat protein is initiated by binding with a RNA stem-loop, resulting in a conformational switch from a symmetric dimer to an asymmetric structure. In this circumstance, detailed structural information was characterized by HDX and MS that some known RNA binding regions showed a more fluctuated HDX kinetics (Morton et al., 2010). PPI dynamics could also be studied by HDX and MS. For instance, Kong et al. (2010) comparatively studied the local conformational rearrangements within HIV-1 gp120 in the presence or absence of CD4. Monroe et al. (2010) also applied HDX combined with LTQ-FT MS to study the immature, mature, and mutant Gag polyprotein to further unravel the capsid assembly.

## Computational Validations by Proteomic Software

With the advent of high throughput short-gun proteomics, liquid chromatography coupled with tandem MS (LC-MS/MS) could characterize numerous fragment ion spectra and thus is able to identify large number of peptide sequences. In addition, accessing highlighted post translational modifications (PTMs) and protein quantifications by stable isotope labeling or label-free analysis have incorporated into the multi-functional search engines, enriching the mass spectrometric data. Presently, several proteomic search engines are available from distinctive searching algorithms and compatible with mass spectrometric data, including some traditional ones, e.g., Mascot, SEQUEST, X!TANDEM, as well as some new search engine, e.g., ProluCID, etc. Mascot incorporates peptide mass fingerprint, sequence query, and MS/MS ions search. It is a possibility-based scoring engine by calculating the observed match between experimental data and theoretical sequence data (Perkins et al., 1999). For instance, Lai et al. (2007) employed Mascot to analyzed the nano LC-MS/MS data and identified up-regulated proteins in human promonocyte cells stably expressing SARS CoV 3C-like protease. Although Mascot provides high throughput protein identification dependent on possibility rankings, there exist some limitations associated with searching non-independent dataset, and non-statistical validation of atypical sequence entries. SEQUEST could correlate ion fragmentations in the processed tandem mass spectrometric data with their corresponding amino acid sequences in the FASTA database files. There are generally four steps of this search algorithm, including tandem mass spectra reduction, matching spectra with amino acids, generating high-ranked sequences, and correlating with protein identification (Eng et al., 1994; Yates, 1998). SEQUEST also is able to search several covalent modification-bearing peptides by matching the nascent tandem mass spectra (Yates et al., 1995). It had been applied to identify secretome of human monocyte-derived macrophages after HIV-1-infection (Ciborowski et al., 2007). X!TANDEM is an open-source platform for proteomic researchers to efficiently process MS/MS data (Craig and Beavis, 2004). Its analysis on a mixture of peptides is based on one axiom: for each detectable protein in the original protein mixture, there will be at least one good tryptic peptide match within a designed scope. In the first step of the analysis, a smaller set of protein sequences is generated from the original protein database by thoroughly filtering with the designed scope that set as small as possible. From this step onward, the subsequent searches are within this refined protein sequences, thus improving the efficiency and saving the overall search time. In the second step of analysis, a bigger scope is set to perform multiple comparisons of the spectra with those refined protein sequences in respect to the different peptide modifications, number of missed cleavages, and non-specific hydrolysis, etc (Craig and Beavis, 2003). Trans-Proteomic Pipeline (TPP) was utilized to statistically analyze the viral and host proteins in purified RSV (Radhakrishnan et al., 2010). And global proteome machine (gpm) is a well-established open-source search engine based on TANDEM^3^. Recently, Xu et al. developed a new search engine ProluCID, which is based on binomial probability preliminary scoring scheme to filter candidate peptides for further isotopic distribution analysis (Xu et al., 2006). And high sensitivity and specificity of ProLuCID could be achieved compared to SEQUEST.

In addition to these searching algorithms, there emerge several multi-functional proteomic pipelines which could incorporate statistical analysis and guarantee high confident searching results. Since different search engines have distinctive algorithms and sensitivities, some important low-abundant hits may be identified by only few software. To cope with this problem, one advantage of Scaffold proteomic pipeline is that peptides simultaneously identified by several different searching engines, e.g., Mascot, SEQUEST, TANDEM, etc, could be integrated into “a folder” by Peptide Prophet Algorithm, resulting in a list of combined peptide sequences. And a further statistical calculation and validation by Protein Prophet algorithm is processed with MS/MS data to generate protein identifications (Searle, 2010). Similarly, TPP also supports the original data generated by Mascot, SEQUEST, TANDEM, etc. Peptide or protein identification is performed by peptide prophet or protein prophet algorithm, respectively. It also combines the advantages of statistical validations by iProphet tool and quantitative analysis by XPRESS, ASAPRatio, or Libra algorithm (Deutsch et al., 2010). Moreover, the Scripps Research Institute developed an integrated Proteomics Pipeline (IP2), which provides a simple and efficient platform for rapid identifications and quantifications to proteomic researchers^4^. It is compatible with both SEQUEST and ProluCID search engines for high resolution MS spectra analysis (Xu et al., 2006). Subsequently, DTASelect set spectrum filtering parameters to ensure low false-positive rate and refine the confidence of protein output (Tabb et al., 2002; Cociorva and Yates, 2007). Census is further incorporated into IP2 to enable large-scale quantitative analysis on isotope-labeled, e.g., N15, SILAC, and iTRAQ, or label-free samples (Park et al., 2008). Some well-established PTMs, especially phosphopeptides, could also be specifically searched during the IP2 ProluCID analysis step, yielding potential highlights for further functional validations.

## Activity-Based Functional Validations

### Limitations of proteomic data

Although the introduction of software is a significant advance to a variety of proteomic studies by performing statistical analysis and filtering out low confident protein candidates, the development of bioinformatics is still hampered by computational limitations. For tandem mass spectra, only a small number of most abundant precursors are generally selected for subsequent MS/MS analysis due to limited sampling rate of MS. It therefore results in missed identification of some lower abundant proteins could escape identification. The method may also identify some non-specific proteins or contaminants, for instance, HSC70 was identified associated with purified RSV by proteomic approach, yet validation by fluorescence microscopy showed that the proteomic observation of HSC70 in RSV was false-positive (Radhakrishnan et al., 2010). Also, for large-scale quantitative proteomic analysis, such as 2D gel and 2D LC-MS/MS, it is hard to validate the functional activity of each protein due to the large quantity of protein IDs and shortages of antibodies. More importantly, mass spectrometric data may also contain proteins from indirect secondary cellular responses that are not directly related to virus infections. For instance, some indirect cellular processes during the late stages of HIV or RSV infection, such as syncytium formation that causes severe secondary host countermeasures could also be detected in proteomic analysis.

**Figure 1:**
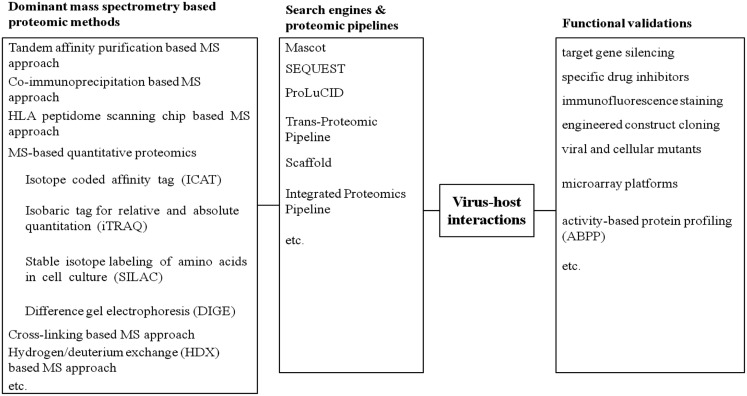
**A summary of current approaches on virus-host interactions**.

To overcome such limitations, more accurate assessments are required to functionally validate the active state of viral or host proteins identified by MS. In this way, functional validations, which highlight proteins involved in direct cellular responses upon virus infections, would provide novel insights to proteomic datasets. These approaches involve drug inhibition of secretory pathway, immunofluorescence staining, dominant negative mutant of protein target, microarrays, small interfering siRNA-mediated knockdown, molecular cloning studies, or activity-based protein profiling (ABPP) (Figure 1). In parallel to our previous review stressing global proteomic studies on virus and host interaction (Zheng et al., 2011), this review mainly focuses on the diverse activity-based applications to validate distinctive proteomic datasets concerning pathogen-host interactions.

### Functional validations by gene silencing

Nucleic acid-based antisense agents have been widely used to inhibit or knockdown targeted gene expression. These antisense technologies constitute antisense oligonucleotides (ODNs), ribozymes, DNAzymes, and RNA interference (RNAi). Each approach has its own strengths and weaknesses. For instance, although there are some off-target effects, RNAi is regarded as a potent and efficient tool for gene silencing even that its sequence target is in low concentration. Also, it could implement *in vivo* and *in vitro* systems, escape the immune responses, and involve in network pathways (Scherer and Rossi, 2003). RNAi is based on the posttranscriptional gene silencing mechanism, which is induced and mediated by small interfering 21–23 nucleotide dsRNA (siRNA; Novina and Sharp, 2004). It targets specific sequence and results in degradation and knockdown of this gene expression. Specifically, natural dsRNAs from the cytoplasm are recognized by RNAi DEfective family member-4 (RDE-4), resulting in dicer-mediated cleavage into 21–23 nucleotide siRNA. These cleaved siRNAs are then recruited into a RNA-inducing silencing complex (RISC), which consists of helicase, exonuclease, endonuclease, and homology searching domains. On one hand, the duplex siRNAs are unwound by helicase. On the other hand, one antisense single strand associated with RISC directs its binding with complementary mRNA. And this binding induces and stimulates the ATP-dependent activities of exonuclease and endonuclease, which could cleave the targeted homologous transcript mRNA (Lee and Sinko, 2006; Pushparaj et al., 2008). Besides natural siRNA, chemically synthesized siRNAs, short hairpin RNA (shRNA), and microRNA (miRNA) could also induce gene silencing in similar mechanisms (Dykxhoorn et al., 2003). A pioneering trial of siRNA was applied to tomato cell lines and antisense 25 nucleotides specific to 1-aminocyclopro-pane-1-carboxylate oxidase (ACO) mRNA were detected (Hamilton and Baulcombe, 1999). In the following 2 years, Elbashir et al. (2001) firstly utilized duplexes of 21-nucleotide siRNAs to mediate the degradation of corresponding mRNAs in mammalian cells. It was also regarded as a breakthrough approach in the year 2002 by *Science* (Couzin, 2002).

In the recent decade, this breakthrough technique has been playing an indispensable role in the realm of virology (Tan and Yin, 2004). In addition, it could integrate and highlight proteomic data by shedding a light at the protein function level. And numerous researches have implemented gene silencing, especially siRNA knockdown, in validating the proteomic data in respect to virus infection. To explore the cellular interacting partners of NS5A of hepatitis C virus, a proteomic technique, Co-IP, was employed (Gonzalez et al., 2009). Heat shock proteins, hsc40 and hsc70, were identified for interacting with NS5A and validated by western blot analysis. NS5A was known to be able to affect the internal ribosome entry site (IRES) mediated translation of HCV. To further determine the roles of these two proteins related to IRES mediated translation, sihsc70 and sihsc40 knockdown combined with a cell culture-based bicistronic luciferase reporter system were designed. The ratio of Firefly to Renilla luciferase expression could reflect the effectiveness of IRES mediated translation. And this ratio is significantly decreased by the knockdown of hsc70, suggesting the importance of hsc70’s role involved in the NS5A alternation of IRES mediated translation. Similarly, Katoh et al. (2011) also used Co-IP purification technique followed by MS to identify the binding partner of flavivirus core protein. Heterogeneous nuclear ribonucleoprotein (hnRNP) A2 was found to interact with flavivirus core protein. By applying siRNA knockdown of this protein, a 90% reduction of viral replication was discovered and vRNA synthesis was delayed, indicating its significant role in regulating virus replications. In another study, TAP based proteomic approach was utilized to explore the interacting complexes associated with viral polymerases of influenza A virus (Jorba et al., 2008). KIAA0136 (NXP2), SFPQ/PSF protein, DEAD/H box polypeptide 3 (DDX3), HNRNP-M protein, coactivator activator, growth regulated nuclear 68 protein (DDX5), beta 5-tubulin, HNRP-H1 protein, ribosomal protein-small subunit S3, and similar to zinc finger protein 71 were specifically identified. Immunofluorescence imaging further confirmed the co-localizations of those host proteins with viral RNPs. And among those identified host factors, a series of functional studies was performed *in vivo* and *in vitro* for characterizing the nuclear associated protein SFPQ/PSF (Landeras-Bueno et al., 2011). siRNA silencing of SFPQ/PSF was found to reduce virus propagations. In particular, the accumulations of vRNA and mRNA were interrupted and reduced; polyadenylation step in viral mRNA assembly was also disturbed when siRNA silencing of SFPQ/PSF was implemented.

To functionally study host proteins associated with virions, siRNA-mediated transcript knockdown is utilized to validate the roles of host proteins involved in virus infection. In one study, highly purified RSV particles were analyzed by proteomic approach, and a total of 25 host proteins were identified to be associated with virions (Radhakrishnan et al., 2010). Among those cellular components, two heat shock proteins, e.g., hsc70 and hsp90, were further selected to examine their effects on virus infection. RSV-infected cells were transfected with either sihsc70 or sihsc90 at 20 hpi. The transfected cells were then fixed and stained with anti-hsc70, anti-hsc90, and antiviral fusion protein (anti-F). In both the sihsc70 and sihsc90 treated groups, obvious reductions in hsc70 and hsp90 staining signals visualized by immunofluorescence imaging were observed, accompanying with decreased viral fusion protein expression. In another study, Spurgers et al. (2010) applied LC-MS/MS and siRNA screening to identify and validate the host proteins associated with Filoviruses (ebola viruses and marburgvirus). The specific knockdown of HSPA5 and ribosomal protein L18 (RPL18) greatly affected ebola virus and marburgvirus infection. qRT-PCR was also applied to confirm the reduced host gene expressions at the transcript level.

For most viruses, the detailed mechanism of virus and host signaling pathways associated with virus survival remains elusive. Since lipid raft plays important roles in virus assembly, signaling, and sorting pathways, delineation of those host proteins associated with lipid rafts could assist to understand the key regulators and mediators involved in viral maturation. Mannova and Beretta (2005) used 2D gel coupled with LC-ESI/Q-TOF/MS-MS to characterize the protein contents of lipid rafts of hepatitis C virus infected cell membranes. N-Ras was identified as a key activator of PI3K-Akt-mTOR pathway and it was important for cellular signaling behaviors. siRNA silencing of N-Ras was observed to inhibit the host PI3K-Akt-mTOR pathway and thus enhanced the HCV replications. A similar effect was observed when cells were treated with PI3K inhibitor LY294002 or transfected with mTOR siRNA. In another study, Gaither et al. (2010) utilized drug inhibition assays, iTRAQ coupled with LC-MS/MS, and RNAi screening to identify key factors involved in HCV replication pathways. Cyclophilins A, H, 40, and E were identified and validated to associate with multiple signaling pathways of HCV replication. One cyclophilin inhibitor NIM811 could strongly suppress these multiple pathways and result in reduced virus release. In another study by Hwang et al. (2007) annexin 1 was discovered to be a key effector to apoptosis pathways associated with infectious pancreatic necrosis virus (IPNV) infected cells. They employed 2D gel electrophoresis and MALDI TOF/TOF MS and identified annexin 1 as an up-regulated protein upon virus infection. SiRNA-mediated knockdown of annexin 1 not only increased the apoptotic effects of cell death, but also suppressed viral protein synthesis until 10 h post infection.

Compared with siRNA knockdown, shRNA and miRNA mediated gene silencing were less reported as functional validation strategies to examine proteomic data. In one study, cellular c-Cbl was known to play a key role in macropinocytosis for entry of kaposi’s sarcoma-associated herpesvirus (KSHV). Valiya Veettil et al. (2010) utilized MS to analyze the immunoprecipitation (IP) of c-Cbl and identified a novel interacting protein, myosin IIA. shRNA mediated knockdown of c-Cbl was then performed and the binding between c-Cbl and myosin IIA was abolished. Overall, these gene silencing studies showed that effective silencing of gene expression has a profound influence to functional virology studies in conjunction with proteomic techniques. What is more, it could provide a more rigorously demonstrated result at the functional level when siRNA-mediated knockdown is comprehensively combined with other approaches, such as immunofluorescence imaging, molecular cloning, RT-PCR, drug inhibition of secretory pathways.

### Functional validations by drug inhibitions

Quantitative proteomic approaches have been frequently utilized to study the relative expression level of viral and cellular proteomes upon virus infection. Precise measurement of expression shift could highlight the groups of significantly up-regulated and down-regulated proteins, which may serve as key mediators or regulators involved in virus-hijacked secretory pathways. Therefore, these significantly regulated proteins characterized by quantitative proteomics could be considered as potential biomarkers or drug targets for diagnostic or therapeutic purposes. In one study, a proteomic approach, 2D gel combined with quantitative analysis was performed to unravel the changes in the cellular proteome before and after dengue virus serotype 2 (DEN-2) infection (Kanlaya et al., 2010a). Sixteen host proteins were found to be up-regulated and twenty two proteins down-regulated. Ubiquitin-activating enzyme E1 (UBE1) was identified as a greatly up-regulated protein and its specific inhibitor, UBE1-41, was selected to explore the effect of UBE1 inhibition on virus propagation and infectivity. The treatment resulted in reduced viral protein synthesis and fivefold decrease in virus release. Therefore the importance of ubiquitin-proteasome pathway to DEN-2 infection could be revealed by specifically counteracting UBE1. Another quantitative proteomic study was designed to illustrate sub-cellular changes upon expression of measles virus nucleoprotein (NP), which is a key mediator involved in cellular apoptosis pathway via triggering reactive oxygen species (ROS) and caspase 3 (Bhaskar et al., 2011). The inhibition of caspase 3 by ascorbic acid could partially reverse and counteract the NP-induced apoptosis. Furthermore, in order to highlight the essential components responsible for antiviral and cell death signaling pathways of influenza A virus infected macrophages, 2D gel coupled with LC-MS/MS was used to quantitatively analyze the up-regulated or down-regulated host proteins in the cytosolic and mitochondrial proteomes (Ohman et al., 2009). As a result, cytoskeleton proteins, e.g., actin and tubulin, were significantly up-regulated in the mitochondrial fraction; and deliveries of certain proteins involved in the antiviral machinery from cytosolic to mitochondrial region were clearly observed. Also, drug inhibition of actin networks by cytochalasin D could impair the expression of certain major antiviral proteins, such as interferon (IFN)-β and TNF-α. These indicate that actin could regulate the antiviral and cell death signaling pathways involved in mitochondrial responses. In another study, as Epstein–Barr virus (EBV) plays a role in gastric carcinogenesis, Fukagawa et al. (2008) used 2D gel followed by LC-MS/MS to quantitatively study the differentially expressed proteins in EBV infected carcinoma cells. Heat shock protein 27 was identified as a significantly up-regulated phosphorylated protein upon virus infection. Drug inhibition studies demonstrated that PI3K/Akt pathway was closely related to Hsp27/phosphorylation, as phosphorylation level was reduced upon treatment with PI3K inhibitors (LY294002 and wortmannin). As a result, suppression of PI3K signaling pathway could inhibit hsp27 function in EBV-induced gastric carcinomas.

Besides Heat shock protein 27, other heat shock proteins are also known to be closely involved in the dynamics of virus and host interactions. Heat shock proteins 40 and 70 were identified by proteomics as binding partners of hepatitis C viral protein NS5A. Gonzalez et al. (2009) used quercetin to inhibit the activities of host factor HSPs upon HCV infection. Both viral replication and release were found to be greatly reduced in a dose-dependent manner compared to a mock group. In another study, heat shock protein 90 (hsp90) was identified to be associated with RSV particles by LC-MS/MS (Radhakrishnan et al., 2010). Immunofluorescence imaging revealed the co-localization of hsp90 with viral filaments and two drug inhibitors, geldanamycin and 17-allylaminogeldanamycin, were used to examine hsp90’s role during virus infection. Viral filamentous staining patterns were greatly impaired while treated with these two inhibitors. Also, the efficiency of virus infectivity and transmission, indicated by estimating numbers of virus infected cells, were reduced by fivefold when treated with these drugs. Although most cellular heat shock proteins function at transcription or replication levels, the low-abundant hsp90 present inside the RSV plays a major role in the formation of virus filaments and virus transmission.

Inhibition of PTM signaling pathways could display therapeutic effects to virus infection, since distinctive PTMs could play different roles in virus infectious cycle. For instance, PB1-F2 is encoded by an alternative open reading frame of the PB1 gene. It is a phosphorylated protein predominantly localized in the mitochondria of some virus infected cells to initiate the apoptosis pathway. Also, PB1-F2 is a virulent factor that it could aggravate virus infection by enhancing the secondary bacterial inflammations (Krumbholz et al., 2011). To explore the innate mechanism of PB1-F2 associated with cellular apoptosis, Mitzner et al. (2009) explored protein kinase C (PKC) mediated-phosphorylation of PB1-F2 by LC-MS/MS and demonstrated the importance of phosphorylation signaling pathways to facilitate apoptosis. Phosphorylation level of PB1-F2 was measured in the presence of inhibitor and activator of PKC, and it is reduced or increased *in vitro*, respectively. When a mutant virus strain with abrogated PB1-F2 phosphorylation sites was produced to infect primary human monocytes, initiation of cellular apoptosis was impaired and virus propagation was reduced. As another example, the spike protein (S) of SARS is a glycosylation-bearing protein, which is important to viral attachment and subsequent fusion steps. Ritchie et al. (2010) utilized the combination of MALDI TOF MS, negative mode Q-TOF MS, and HPLC to comprehensively study the various types of N-linked carbohydrates associated with the viral spike protein. Major complex glycans were clearly characterized. And treatment of SARS infected Vero E6 cell with α-glucosidase inhibitor *N*-butyl-deoxynojirimycin (NB-DNJ) revealed that the suppression of glycosylation process could inhibit the virus infection by blocking the initial stage.

Drug inhibitions specifically targeting viral proteins have been increasingly in demand to block virus infections. However, some viral proteins, e.g., hemagglutinin (HA) and neuraminidase of the influenza A virus, could exhibit their remarkable potentials to mutate to new subtypes. Thus it is necessary to require an updated drug discovery strategy to cope with prophylaxis upon virus infection. Clinically, there are two available drugs against neuraminidase: zanamivir and oseltamivir (Shahrour, 2001). Oseltamivir, also known as tamiflu, was the first orally active neuraminidase inhibitor able to defend the H5N1 avian flu and H1N1 swine flu in early infection (Agrawal et al., 2010). And zanamivir is effective to prevent symptomatic laboratory-confirmed influenza virus and its transmission in healthy adults (Jackson et al., 2011). In addition, adamantine is widely used to block the Matrix 2 (M2) ion channel in vaccine and antiviral drug designs. Nevertheless, both adamantine-resist and tamiflu-resist circulating influenza A virus strains were emerged in the last century (Deyde et al., 2007; Hayden and de Jong, 2011; Sheu et al., 2011). Therefore, a strategy for updated drugs or vaccines targeting new viral mutants should be urgently established in preparation for the next pandemics or epidemics. The utility of proteomics has been efficiently employed to facilitate the vaccine design and antiviral drug discovery. For example, the binding between influenza HA and α-2, 6-sialylated glycoprotein receptor is the initial step to induce human infection. Also, the HA precursor is further cleaved by proteases on the virus surface to generate the C-terminal fragment HA1 and the N-terminal fragment HA2, triggering the potential ability of membrane fusion process (Wiley and Skehel, 1987). Thus, HA or its binding with sialic acid receptors could be regarded as potential drug targets to block the initial stage of virus infection. To characterize the innate immunity antiviral components from salivary, Chen et al. purified the binding partners of α-2, 6-sialylated glycoprotein receptor. And α-2-macroglobulin (A2M) was identified by proteomic approach for specifically inhibiting hemagglutination (Chen et al., 2010).

### Functional validations by immunofluorescence staining

Immunofluorescence staining assay is a powerful tool which combines the utility of specific fluorescent probes, advanced confocal microscopy, and digital image analysis. It has been frequently used for analyzing distinctive biological samples, such as neurons (Zinchuk and Grossenbacher-Zinchuk, 2009), plant cells (French et al., 2008), virus infections, and so on. Co-localization refers to the co-existence of multiple fluorescent probes generated through different fluorochromes, resulting in overlapped images. For instance, in a same specimen, two antigens are visualized by their respective fluorescence-labeled secondary antibodies in microscopy (one is red while the other is green), and a third yellow staining image emerges when these two antigens co-localize, suggesting the interactions of these two macromolecules or particular sub-cellular localizations where this co-existence belongs. However, there also exist some limitations of this technique (Smallcombe, 2001). For example, fluorescence bleed-through and tissue autofluorescence could take place between two fluorochromes, thus resulting in increased non-specific background noises. These obstacles could be overcome by careful sample preparation and appropriate optimization of image acquisition (Smallcombe, 2001; Zinchuk et al., 2007). In addition, the bimolecular fluorescence complementation (BiFC) assay could investigate PPIs in live cells and organisms (Kerppola, 2006, 2008). Two fluorescent protein fragments are fused to target proteins that interact, and these fragments would refold to produce fluorescence upon target protein association. BiFC has been utilized to study the virus-host interaction between HSV-1 regulatory protein ICP27 and one cellular protein, TAP/NXF1 (Hernandez and Sandri-Goldin, 2010). Yet few works were present as a subsequent method to validate proteomics data related to virus infections.

Co-localization has been widely applied to virology studies in order to visualize the co-existence of target proteins in different sub-cellular distributions. In proteomic studies, the confocal imaging technique could further visualize virus-host interactions at the protein level and thus enhance proteomic data from another aspect. In one research, Lee et al. (2011) employed TAP based pull-down assay combined with 1D LC-MS/MS to investigate the essential interaction partners of hepatitis C virus core (HCVc) protein. Huh7 cells were transiently transfected with a plasmid construct bearing exogenous HCVc and TAP components, which were designed to express the biotinylated (B-tag) bait protein with calmodulin-binding peptide (CBP)/protein A tags. This TAP based proteomic approach enabled the authors to identify 36 candidates. Three highest-ranking proteins, hnRNPH1, NF45, and C14orf166, were selected for further validations. These three proteins were also specifically identified and validated as binding partners of HCVc in another affinity pull-down system, which is based on streptavidin-Dynabeads instead of IgG-Dynabeads. Confocal imaging analysis was performed to visualize these interacting partners. In a mock group, hnRNPH1 was found to be localized in the nucleus whereas NF45 and C14orf166 were evenly distributed in both the cytoplasm and the nucleus. In HCVc expressed Huh7 cells, co-staining of HCVc with these three proteins were predominantly localized in the nuclear region, suggesting that HCVc was transferred to nucleus from cytoplasm to interact with its binding partners. In another example, an EBV-encoded protein, latent membrane protein 1 (LMP1), was known to be able to interact with cellular prenylated Rab acceptor 1 (PRA1). This association mainly occurred in Golgi apparatus visualized by immunofluorescence imaging and it was highly involved in LMP1 mediated intracellular trafficking and NF-kB signaling pathways in nasopharyngeal carcinoma (NPC) cells (Liu et al., 2006). To further clarify the role of PRA1 in EBV infected NPC cells, siRNA knockdown of PRA1 was performed to generate PRA1-knockdown NPC clones, which were analyzed by the isobaric mass tags (iTRAQ) labeling approach coupled with 2D LC-MS/MS (Liu et al., 2011). Seventy proteins were found to be significantly up-regulated whereas 20 were down-regulated. The significantly up-regulated proteins (e.g., LAMC2, ITGA6, ITGB4, FABP5, CAV1, and TIP47) responsible for lipid homeostasis and cell migration were selected and analyzed by immunofluorescence imaging in PRA1-knockdown NPC cells. In consistent with the proteomic data, spatial distributions of ITGA6, ITGB4, and CAV1 to perinuclear regions were observed with their elevated fluorescence staining patterns.

In another study associated with dengue virus infected human endothelial cells, Alix (apoptosis-linked gene-2-interacting protein X) was identified as an up-regulated protein by 2D gel coupled with Q-TOF MS/MS (Pattanakitsakul et al., 2010). Alix was known to play an important role in viral protein transport from endosome to cytosol for viral replication purpose. And double immunofluorescence staining assay confirmed the co-localization of Alix with the late endosome marker, lysobisphosphatidic acid (LBPA), suggesting that interaction of Alix with late endosome could assist the viral nucleocapsid export to the cytoplasm. Also, cells with and without anti-LBPA pretreatment were infected with dengue virus and then measured in different time post infection; immunofluorescence staining by targeting DENV envelop protein revealed delayed virus replication when pre-treated with anti-LBPA, suggesting that the inhibition of endosomal protein could impair functions of Alix to assist viral replication. To explore the possible mechanism underlying vascular leakage upon dengue infection, a similar proteomic approach was utilized to quantitatively analyze significantly regulated proteins (Kanlaya et al., 2009). β-actin was identified to be greatly up-regulated. However, subsequent western blot analysis revealed that the expression level of β-actin was decreased. Furthermore, by applying immunofluorescence imaging, dengue infected cells showed remarkably decreased expression of actin networks and proteins associated with adherens junction, intercellular adhesion, and transendothelial migration. These observed alternations of actin networks and endothelial integrity could shed a light on vascular leakage upon virus infection. Although β-actin was noted as an up-regulated protein in one particular spot by 2D Gel proteomic approach, it was possible that the overall expression level of this abundant protein was decreased, which was consistent with the western blot and immunofluorescence imaging assays. This observation also reflects the necessity of functional validations to proteomic data, which may include false-positive identification or biased quantification results. Meanwhile, due to the limitations of the 2D gel based proteomic approach (Bunai and Yamane, 2005), its accuracy needs to be improved. Besides β-actin, hnRNPs were also found to be up-regulated upon virus infection, and IP based proteomics identified vimentin as the binding partner of hnRNPs (Kanlaya et al., 2010b). The subsequent co-localization study confirmed the co-existence of vimentin, hnRNPs, and dengue NS1 in perinuclear regions, suggesting their roles associated with assembling in perinucleus upon dengue virus infection. The advances of immunofluorescence staining in visualizing the sub-cellular localizations of specific proteins and their interactions ensure that it remains a vital technique for functional proteomic studies.

### Functional validations by construct cloning

Construction of plasmid cloning vector encoding gene targets and its subsequent transfection into cells, or bacteria, could lead to the co-expression of endogenous and exogenous proteins. Therefore, this target-based cloning enables us to additionally induce the over-expression of one protein or heterogeneously express proteins encoded by genes of an alternative microorganism, respectively. In recent years, numerous pull-down based proteomic techniques have been widely applied to study PPIs. Specifically, TAP based proteomic approaches facilitate the characterization of interacting partners of the highlighted viral protein, which could be exogenously expressed in host cells by cloning specific construct. And Most TAP based or pull-down proteomics were initial designs followed by proteomics rather than functional validation studies to proteomic data. In one study, in order to discover the binding partners of HCVc fusion protein, Lee et al. (2011) designed a vector construct by inserting the HCVc gene fragment into the pMSCV-BCP vector, this enabled them to express a biotinylated (B-tag) bait protein with CBP/protein A tags. The interacting protein complex of the exogenous intact HCVc protein could be identified by LC-MS/MS and confirmed by confocal imaging microscopy. In another study, Jorba et al. (2008) constructed plasmids encoding influenza A viral polymerase genes fused with the TAP tag, and used MALDI TOF MS to find the binding partners of this heterotrimeric polymerase complex. Human HEK293T cells were transfected with these plasmids expressing viral polymerase proteins fused with the TAP tag. Most successfully identified host proteins were nuclear proteins involved in cellular RNA synthesis, modification, and nuclear trafficking. To further validate the proteomic data, one abundant host factor, KIAA0136 (NXP2), was designed to be over-expressed from the plasmid pcDNA3HA-NXP2 by fusing the gene with the HA tag. And subsequent immunofluorescence imaging assay supported mass spectrometric data and confirmed the co-localization of this tagged-NXP2 with viral RNPs.

What is more, pull-down based proteomic method could also be applied to investigate interacting partners of viral genomes, e.g., HIV tat/rev exon (Marchand et al., 2011), and thus illustrate virus replication mechanisms. The 5′ and 3′ ends of transmissible gastroenteritis virus (TGEV) genome were known to harbor *cis-acting* signals and have affinity preference for host binding partners. Galan et al. (2009) synthesized two respective TGEV genome ends containing first the 504 nucleotides or the last 493 nucleotides by PCR and labeled them with biotin prior to *in vitro* transcription. The biotin labeled RNA were then immobilized on a streptavidin sepharose resin as baits for affinity protein purification, followed by MALDI TOF/TOF MS identification. Nine proteins displayed preferential binding to the C-terminal of the viral genome whereas one protein was found to interact with the N-terminal. And siRNA knockdown of these C-terminal interacting proteins, e.g., PABP, hnRNP Q, and EPRS, resulted in significant reduction in viral RNA synthesis, suggesting their roles associated with viral transcription and replication. In a similar study, KSHV is a DNA virus and its terminal repeat (TR) genome elements could potentially interact with cellular components during virus infection (Si et al., 2006). A triple copy of the 801 bp TR was inserted into a plasmid vector, pBSpuroA3, which could be transfected and amplified in a KSHV-negative cell line and a KSHV-positive cell line. The TR elements associated with cellular factors were digested with corresponding restriction enzymes and purified with affinity column prior to proteomic analysis. Some candidate proteins identified by proteomics (PARP-1, ATR, NPM1, and BRG1) were further corroborated by western blot and co-localization studies. Analogously, Lin et al. (2008) also utilized RNA affinity poll down and proteomics to study protein interacting partner of the 5′ untranslated region of enterovirus 71, and identified hnRNP K. Plasmids encoding different truncated forms of hnRNP K were further designed to specifically locate interaction domains. KH2 and the proline-rich domains were affirmed by western blots and siRNA knockdown of these domains resulted in decreased viral yields and viral RNA synthesis. In addition to RNA precipitation coupled with MS, another proteomic technique, reversible formaldehyde cross-linking coupled MS is able to map the specific peptides that bound to viral RNA (Kim et al., 2005). Viral replicase of potexviruses has a helicase domain, the cDNA of which was cloned into pET32 fused with thioredoxin, a His tag, and an S tag at its N-terminal. This enzymatic protein was expressed in *E. coli*, and purified by immobilized metal affinity chromatography. Subsequently, this purified protein was incubated with a 3′- biotinylated 15-nt RNA in the presence of formaldehyde, a cross-linking agent. After trypsin digestion, peptides bound to RNA were purified by IP, followed by LC-MS/MS analysis. A total of six peptides were identified by proteomics. Their RNA binding affinities were validated by functional mutation analysis.

In addition, quantitative proteomics could reveal virus-host interactions with distinctive profiles after plasmid transfections. The X protein of chronic hepatitis B virus (HBx) was known to induce hepatocellular carcinoma (HCC). In one study, HBx of genotype A, B, C were amplified by PCR and inserted into pXJ40 vector, followed by transfection HepG2 cells (Feng et al., 2010). Cells transfected with those different genotypes of HBx and empty pXJ40 plasmid were labeled with iTRAQ and further submitted to 2D LC-MS/MS. Comprehensive protein profiling displayed some up-regulated cytoskeleton proteins responsible for cytoskeleton movement and migration. For instance, microtubule-actin cross-linking factor 1 (MACF1), annexin A2, high mobility group box 1 (HMGB1), were over-expressed after HBx transfection. To further functionally validate the proteomic data in respect to cellular motility, the HepG2 cells were co-transfected by those HBx genotypes and green fluorescent protein (GFP) to visualize the tracks of cell movement by real-time fluorescence microscopy. And HBx genotype A infected cells revealed more vigorous movement than cells infected with other HBx genotypes. Similarly, Mota et al. (2008) utilized quantitative proteomics to characterize the differently expressed protein profiles upon the presence of differentially viral components of hepatitis delta virus (HDV). Huh7 cells were transiently transfected with plasmids coding S-HDAg, L-HDAg, gRNA, and agRNA, respectively, and changes in the Huh7 cell proteome were measured by 2D gel and MALDI TOF/TOF MS. Among those differentially expressed proteins, hnRNP D, HSP105, and triosephosphate isomerase were down-regulated and confirmed by RT-PCR. This work was the first to present the overall host alternations upon expression of HDV proteins and genes, thus providing clues to unravel detailed HDV replication mechanisms. Similar approaches have been used to quantify the proteome changes upon expression of exogenous small hepatitis B surface antigen (SHBs) in HepG2 cells (Zhao et al., 2010), recombinant influenza viral protein fused with HIV-1 p17 protein in CD8+ T cells (de Goede et al., 2009).

### Functional validations by mutants

Viruses associated with carcinomas and tumors have presented high risks and occupied an important branch of cancer diagnostics. Deciphering virus and host interplays by Co-IP based proteomics could gain tremendous insights into malignant transformations of carcinomas as well as cellular differentiation and proliferations encountered. In addition, studying the effects of those negative mutants could greatly enhance the validity of proteomic data. Adenovirus (Adv) was a DNA virus model suitable for studying tumor oncogenesis, Komorek et al. (2010) successfully utilized TAP based proteomics to identify multiple cellular proteins interacting with the Adv oncoprotein E1A C-terminal region. The forkhead transcription factors, FOXK1/K2, were identified as novel factors specifically bound to E1A. Numerous recombinant adenoviruses carrying distinctive E1A exon 1 and exon 2 mutants were generated in order to map the specific E1A domain interacting with FOXK1/K2 by western blot analysis. One mutant lacking amino acids 224–238 in exon 2 was found to have significantly reduced staining with FOXKI/K2. Functionally, virus bearing this E1A exon 2 mutant was deficient in association with FOXK1/K2, resulting in enhanced cell proliferation and oncogenic transformation. Thus this indicated that the interaction between FOXK1/K2 with exon 2 of E1A could reversely suppress cell proliferation and oncogenic transformation. Human Papillomavirus (HPV) is another DNA virus causing anogenital carcinomas and oropharyngeal squamous cell carcinomas. The viral gene E7 is retained and integrated into the cancer cell chromosomes. Therefore its E7 oncoprotein plays important role in mediating malignant transformation. In one study, in order to find the novel binding partners of the E7 protein, a recombinant E7 from HPV-16 was constructed by tagging *S. japonicum* GST to its N-terminal, which could be recognized by immobilized glutathione. Human glutathione *S*-transferase P1-1 (GSTP1) was uniquely identified by MS. The interaction between E7 and GSTP1 was structurally characterized by three-dimensional docking program, which assisted to design a E7 mutant deficient in affinity binding with GSTP1 by subtracting residues Val 55, Phe 57, and Met 84. Although real-time PCR showed similar translation levels of GSTP1 in E7 and mutant group, GSTP1 in HPV E7 expressing cells apparently enhanced cell pro-survival abilities by suppressing Jun N-terminal kinase (JNK) mediated-phosphorylation signaling pathway to induce apoptosis. And siRNA knockdown of GSTP1 in HPV E7 expressing cells could reversely counteract this effect (Mileo et al., 2009).

Viral proteins important for manipulating viral gene expressions could be post-translationally modified, playing essential role as intrinsic functional proteins and cofactors assisting or inhibiting virus infection. Validations with viral PTM-deficient proteins by substituting PTM sites enable us to make a comparative study with the control group, uncovering the impact of these modified proteins at the functional level. For instance, ICP27 is one viral regulatory protein of herpes simplex virus type 1 (HSV-1). It is known to be post-translationally modified by kinases and arginine methyltransferases, and closely involved in viral protein export (Sandri-Goldin and Hibbard, 1996). ICP27 bears a glycine- and arginine-rich (GAR) region within an RGG box, which is characterized as an RNA binding domain mediating viral protein export. Souki et al. employed Co-IP approach to purify ICP27 in virus infected cells and performed independent trypsin, pepsin, and thermolysin digestion to comprehensively map the sequence of ICP27 by MALDI TOF/TOF MS (Souki et al., 2009). As a result, this combined protease digestion method was able to detect peptides covering of 90% of the ICP27 sequence, including the major arginine methylation sites, e.g., arginines 138, 148, and 150 within the RGG box. To functionally study the arginine methylation associated with virus infection, site-directed mutagenesis was used to construct point mutation (arginine to lysine) in ICP27, which was inserted in a plasmid and co-transfected with viral DNA into cells. The R150K mutant exhibited the most delayed virus maturations as well as smallest plaque size. Also, microarray assay revealed that both viral gene expression and replication were reduced in the mutants compared to the wild type, suggesting that the role of arginine methylation of ICP27 is closely associated with virus trafficking, assembly, as well as virus genome transcriptions. Similarly, phosphoprotein (P) of parainfluenza virus 5 (PIV5) was heavily phosphorylated and it was responsible for viral RNA synthesis upon infection (Sun et al., 2011b). IP was carried out to bait the viral P protein by using immobilized anti-V5-conjugated agarose, followed by SDS-PAGE gel and LC-MS/MS analysis. T286 of the P protein was found to be phosphorylated and it was further substituted by alanine, aspartic acid, or glutamic acid to study the effects of P mutants to viral infection. Compared to the wide type, the virus carrying the mutant P protein T286A was observed to have a slower growth rate as well as delayed viral mRNA synthesis at the transcription level. This work demonstrated that phosphorylation of the PIV5 P protein is important for virus replication and formation; however the detailed mechanism of key kinases involved in signaling pathways remains elusive. In another study, Duellman et al. (2009) used immobilized metal-affinity chromatography (IMAC) to enrich the phosphorylated peptides of EBV nuclear antigen 1 (EBNA1) followed by nano LC-MS/MS analysis, which resulted in identification of 10 phosphosites on EBNA1. In the subsequent validation works, all the 10 phosphosites were mutated to alanine by PCR mutagenesis and constructs were amplified in stable 293T cells after transfection. The phosphorylation of EBNA1 was essential to virus transcriptions as its phosphorylation-deficient mutant revealed a reduction on transcription activities.

What is more, some host proteins could also balance their distinctive PTM sites, and therefore manipulate cellular immune responses upon virus infections. Retinoic acid-inducible gene I (RIG-I) protein is one cytosolic receptor sensitive to viral RNAs. And this recognition could further undergo ubiquitination at Lys72, which enables to induce the innate immune responses, e.g., type-I IFNs, to inhibit the viral replication. Whereas under the normal condition, some kinases could phosphorylate RIG-I and thereby inhibit ubiquitination and downstream antiviral signal transduction. To map the phosphorylation sites on RIG-I, Gack et al. constructed the GST fusion vector inserted with the RIG-I PCR product, which was expressed in HEK293T cells (Gack et al., 2010). By employing GST pull-down and LC-MS/MS analysis, three phosphorylation sites in the N-terminal caspase recruitment domains (CARDs) of RIG-I were identified. It was hypothesized that phosphorylation at T170 suppresses ubiquitination at Lys172. The T170E mutant was generated to explore this hypothesis under normal circumstances, the phosphorylation level of RIG-I was markedly reduced after virus infection whereas its ubiquitination level was increased in a time-dependent manner. However, the T170E mutant lacked binding ability with tripartite motif protein 25 (TRIM25), which could induce ubiquitination and IFN signal transduction. Furthermore, Maharaj et al. (2012) successfully identified two upstream kinases, PKC -α and C-β that are responsible for phosphorylation of the RIG-I protein. Double knockdown of PKC- α/β by shRNA or siRNA revealed remarkably decreased phosphorylation levels, resulting in increased susceptibility of cells to virus infection.

### Functional validations by microarray platforms

In addition to MS based proteomics, protein microarrays have also been considered as a set of rapidly evolving technologies capable of identifying PPI networks, quantitatively profiling protein expression levels, and complementing the high throughput proteomic data (Sobek et al., 2006; Pollard et al., 2007). In the recent decade, much effort has been devoted to systematically study biochemical activities of proteins in a high throughput manner. Yet the major difficulties are screening an entire proteome by expressing clones, accommodating low volume proteins, and meanwhile retaining biochemical activities. Zhu et al. (2001) cloned 5800 open reading frames and purified corresponding proteins in order to construct a yeast proteome chip. GST-labeled proteins were immobilized onto the glass microscope slides through covalent attachment by using aldehyde-amine or nickel-HisX6 tags. In a parallel study, microarrays containing thousands of Cy3 or Cy5 labeled proteins were fabricated by a high-precision contact-printing robot to generate nanoliter protein spots on glass slide. Its applications, such as protein-small molecule and kinase-substrate interactions were successfully verified (MacBeath and Schreiber, 2000). In recent years, both proteomics and protein microarrays are predominantly used as reliable tools in glycomic profiling with a screening effect (Mahal, 2008). Characterization of protein-glycan interaction related to virus infection is another important task in the field of glycoproteomics and glycan microarrays. For instance, the binding specificity of influenza A virus with sialic acids is the main determinant for virus entry and species-dependent infection. To discover the detailed glycan-binding preference for the pandemic “triple reassortant” influenza A virus and a low infectious influenza A virus, Bateman et al. (2010) utilized MALDI TOF MS and GC-MS/MS to extensively map the linear or branched, N- or O-linked glycans expressed on the surface of primary swine respiratory epithelial cells (SRECs). Different sialic acid linkages, such as α-2, 3 or α-2, 6 linkage, were also determined by MS after corresponding sialidase digestions. Both NeuAc and NeuGc were identified on the SRECs surface with NeuAc occupying a much higher abundance. By applying glycomic microarray analysis, it revealed that both virus strains were ready to bind to NeuAcα2-6 glycans, sialylated polylactosamine and sialylated *N*-glycans. Therefore, the structural characterization of a wide variety of glycans by proteomics and functional microarray based on HA-sialic acid interactions could expand our knowledge on the molecular basis of virus-host interactions. In a similar study, Song et al. (2011) generated a total of 77 α-2, 3 or α-2, 6-sialylated structures incorporating different types of modified or nature sialic acids. All these sialylated glycans were examined by MALDI TOF MS and fabricated on a NHS-activated microarray glass slide. Several human influenza viruses, such as H1N1, H3N2 were tested on this glycan microarray, and both viruses were able to bind specifically to α-2, 6 linked sialic acid derivatives, α-2, 6 linked Neu5Ac, and α-2, 6 linked Neu5Ac9Lt. H1N1 displayed a broader range of binding specificities than H3N2 with an additional binding to α-2, 6-sialylatedNA2 structures. In this way integrated with proteomics, microarray screening of glycan-pathogen interactions with a wide variety of sialic acids provides an efficient way to explore glycan recognition at the molecular level.

What is more, these emerging large-scale screening technologies have tremendous impact on deciphering pathogen-host interplays. For instance, antiviral immune responses by production of type-I IFNs upon virus infection are regulated by TANK-binding kinase 1 (TBK1) and I-kB kinase ε (IKKi). TBK1 was observed to play a more profound role than IKKi (Hemmi et al., 2004). To understand the molecular interacting network of TBK1/IKKi involved in regulating IFNs upon virus infections, Goncalves et al. employed TAP based MS to study interacting partners of TBK1, IKKi, and their corresponding adaptor proteins, TANK, Sintbad, and NAP1. Consequently, MS identified 30 binding partners of these five proteins, representing a small interaction network of TBK1 and IKKi. Using RNA based microarray assay, overall changes at the transcription level were illustrated by intensity in response to virus infection or poly (I:C) simulation, and it was found that TBK1-TANK binding contributed greatly to the TBK1 activation (Goncalves et al., 2011). Clinically, high throughput proteome microarray bearing antigens of HPVs was able to examine immune responses and antigenicity of HPV proteins in a vast number of patients’ serum samples (Luevano et al., 2010).

### Functional validations by activity-based protein profiling

Although MS based proteomics has high throughput for identification of the altered proteome upon virus infections, it is unable to characterize the dynamic changes of enzymes, which could be induced by distinctive modifications, proteolytic processing, and alternative regulatory proteins. ABPP is a common functional technique designed to measure the dynamic changes of enzyme activities during virus infections (Cravatt et al., 2008; Blais et al., 2012). The activity-based probes (ABPs) consist of two essential components: a warhead reactive group and a reporter tag, which are designed to covalently target the active site of enzymes and for purification or visualization, respectively. In addition, different levels of enzyme activity induced by viral or host proteins upon virus infections could be studied by comparative ABPP. A non-directed ABPP probe, PS4, was used to profile different levels of enzyme activity related to HCV replications in Huh7 cells (Singaravelu et al., 2010). Nine host candidates such as HSPA8, protein disulfide isomerase A5, and nuclear distribution gene C homolog were identified by MS based on comparative ABPP analysis. What is more, a FP-rhodamine ABPP probe was used to examine the host serine hydrolases required for HCV replication (Blais et al., 2010). After 2D gel, protein spots recognized by the FP-rhodamine ABPP probes were visualized by fluorescence prior to LC/MS/MS analysis. Carboxyl-esterase 1 (CES1) was identified as a differentially active enzyme involved in regulating triglycerides and cholesterol. siRNA knockdown of CES1 further resulted in reduced HCV replication levels and over-expression of CES1 benefited virus replications. To explore different activities of ubiquitin specific proteases under different pathological conditions, Ovaa et al. used HA-tagged Ub-derived active-site-directed probes to comparatively study the enzyme activities in healthy, virus infected, and tumor-derived cells. The altered USPs were purified and followed by LC/MS/MS analysis, resulting in identification of a list of up-regulated USPs in different stages of cellular differentiation (Ovaa et al., 2004). Furthermore, ABPP could also serve as a novel tool for discovering selective drugs or inhibitors. For instance, tetrahydroquinoline oxocarbazate was characterized as a blocker against SARS coronavirus and Ebola pseudotype virus by inhibiting cathepsin L, a member of human lysosomal cysteine proteases (Shah et al., 2010). Therefore, this breakthrough technology demonstrated its advantages by functionally identifying enzyme activities associated with a wide range of diseases, as well as filling up the gap that are unreachable to MS based proteomics.

## Conclusion

Currently, challenging problems arise when the ever-expanding repertoire of diverse viral proteomes leaves a large number of viral proteins uninvestigated or even larger numbers of virus-host interactions functionally unverified. It relies heavily on high throughput technologies to elucidate the pathogenic biological networks involved in virus infections. At the interface between the realms of virology and proteomics, the conjunction of MS based proteomics with functional validation approaches exhibits a multidisciplinary effort to enhance our understanding into a wide range of viral diseases. These activity-based validation approaches integrated with proteomics include gene silencing, immunofluorescence staining, molecular cloning, drug inhibition, and microarrays, etc. Thus, it is important to stress that functional validations play an indispensable role to studying protein activities that are inaccessible to proteomic data along, contributing enormously and consistently to our understanding of pathogen-host interactions, vaccine designs, biomarker explorations, and drug discoveries.

## Conflict of Interest Statement

The authors declare that the research was conducted in the absence of any commercial or financial relationships that could be construed as a potential conflict of interest.

## Footnotes

^1^http://www.ncbi.nlm.nih.gov/genomes/GenomesGroup.cgi?taxid=10239&opt=Virus

^2^http://www.uniprot.org/taxonomy/?query=virus+AND+reviewed%3ayes&offset=250

^3^http://www.thegpm.org/

^4^http://integratedproteomics.com/
